# Diastema Closure in Anterior Teeth Using a Posterior Matrix

**DOI:** 10.1155/2016/2538526

**Published:** 2016-10-13

**Authors:** Ayush Goyal, Vineeta Nikhil, Ritu Singh

**Affiliations:** ^1^Department of Conservative Dentistry & Endodontics, Subharti Dental College, Meerut, Uttar Pradesh, India; ^2^Department of Paediatric and Preventive Dentistry, Subharti Dental College, Meerut, Uttar Pradesh, India

## Abstract

Presence of diastema between anterior teeth is often considered an onerous esthetic problem. Various treatment modalities are available for diastema closure. However, not all diastemas can be treated the same in terms of modality or timing. The extent and the etiology of the diastema must be properly evaluated. Proper case selection is of paramount importance for a successful treatment. In this case report, diastema closure was performed with direct composite restorations. One bottle etch-and-rinse adhesive was used and a single shade was used to close the diastemas. Contoured sectional posterior matrix was used to achieve anatomic contouring of the proximal surfaces of the teeth. This was followed by finishing and polishing using polishing discs. Patient was kept on recall every 6 months.* Conclusion*. Diastema closure with correct anatomic contouring is easy to perform using the contoured sectional matrices. At 14-month recall, no clinical signs of failure like discoloration or fracture were evident. Also, patient did not complain of any sensitivity. Thus, direct composite restorations serve as durable and highly esthetic restorations leading to complete patient satisfaction.

## 1. Introduction

Midline diastema is defined as anterior midline spacing greater than 0.5 mm between the proximal surfaces of central incisors [1]. Midline diastema or spacing in anterior teeth is a common condition that can present itself anytime to the dental office. It has been reported that maxilla has a higher prevalence of midline diastema than mandible [2]. Midline diastema is multifactorial in etiology. Some of the causes include maxillary incisor proclination, labial frenum, incomplete coalescence of the interdental septum, pseudo-microdontia, presence of a mesiodens, peg-shaped lateral incisors, congenital absence of lateral incisors, pathologies (e.g., cysts in the midline region), habits such as finger sucking, tongue thrusting, and/or lip sucking, discrepancy in the dental and skeletal parameters, and probably genetics [3].

Once the etiology is known, a decision must then be taken whether to utilize a multidisciplinary approach or to simply close the spaces by means of direct and/or indirect restorative treatment. If the teeth are correctly aligned and positioned, but the tooth size is the culprit, the clinician is left with the task of selecting the best restorative procedure [4].

The development of composite resins with superior mechanical properties and excellent polishability allows the clinician to mimic the natural dentition and render a long-lasting restoration to the patient. Also, composite resins permit conservative treatment and at the same time offer quicker results [5].

Recent aesthetic composite resin materials have similar physical and mechanical properties to that of the natural tooth and possess an appearance like natural dentin and enamel. They offer a diverse range of shades and varying opacities designed specifically for layering technique [6, 7].

Creating an anatomic contour without “black triangles” is an arduous task when closing diastemas using resin-based composites. This case report describes diastema closure in the maxillary anterior region using resin-based composite material with the help of stainless steel precontoured matrices.

## 2. Case Report

**Figure 1:**
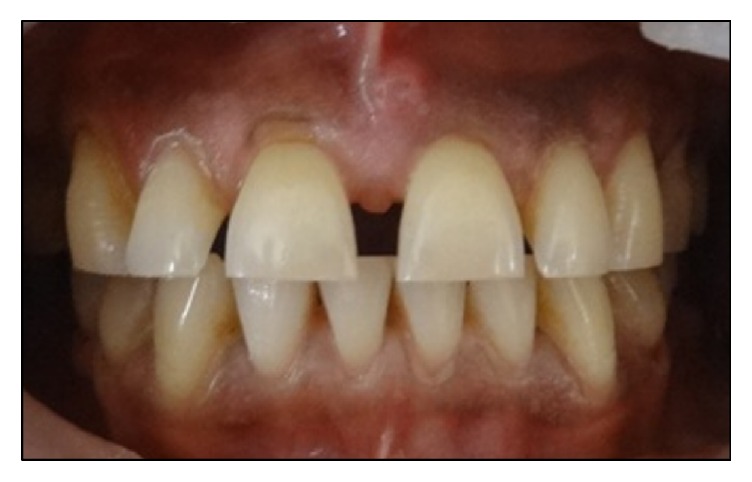
Preoperative intraoral view of the patient shows interdental spacing in maxillary anterior regions.

**Figure 2 fig2:**
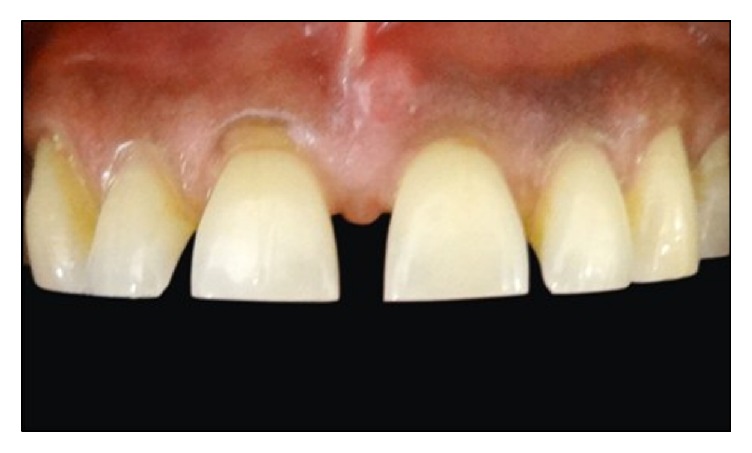
Preoperative intraoral view of maxillary anterior regions.

**Figure 3:**
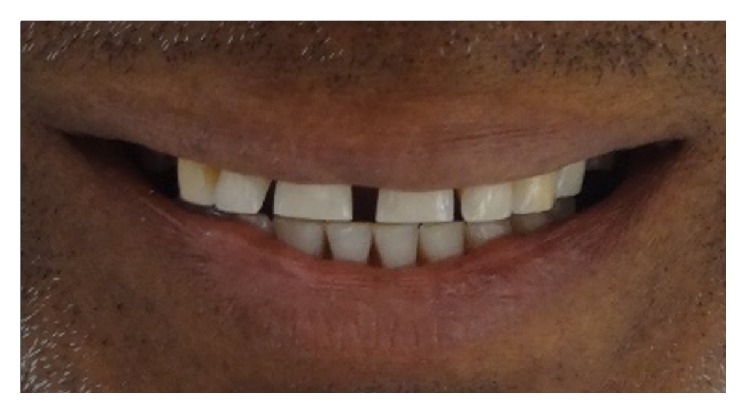
Preoperative extraoral view of the patient.

A 52-year-old male patient reported to the department of Conservative Dentistry and Endodontics, Subharti Dental College, with a chief complaint of spacing in his upper front teeth region. Patient's medical history was noncontributory and intraoral examination using a Vernier Caliper (Aerospace Digital Vernier Caliper, India) revealed interdental spacing between maxillary central incisors (~4 mm) and maxillary central and lateral incisors (~1.5 mm) (Figures 1 [Fig fig2]–3). No dental caries were observed upon both clinical and radiographic examinations.

The patient was satisfied with the color of his teeth. He had a thick gingival biotype and a fairly symmetrical gingival architecture. Patient demonstrated good periodontal health upon clinical examination. Plaque Index [8] was used for periodontal evaluation. The score obtained was 0.57 which signifies good oral hygiene.

Among the different treatment options for this case, we selected the most conservative because of patient's desire for quick results and his financial constraints. Also, though not a regular visitor to the dentist, the patient demonstrated good oral health.

Before starting the treatment, preoperative photographs (Nikon® Coolpix L810) were taken. Following oral prophylaxis, shade selection was done using the VITAPAN® Classical Shade guide (A2) and an intraoral mock-up was done with A2B shade of Filtek™ Supreme XT (3 M/ESPE, St. Paul, MN, USA) but without etching and bonding. Two bulk increments of the composite were placed on the mesial surfaces of 11 and 21 and gross contouring was done with a composite instrument. Then, the composite was cured only for 20 seconds and the outcome was shown to the patient. Since etching and bonding procedures were not done, the bulk of composite restorative material could be easily removed using a sharp instrument. Once the patient was satisfied, it was decided that only a single shade (A2B) would be used to close all the diastemas. The midline diastema was closed by building up the mesial surfaces of central incisors one by one. It was decided to restore 21 first. No tooth preparation is necessary prior to adhesive procedures. Roughening of the enamel is recommended only when self-etch adhesives are to be used. 37% phosphoric acid (Etching Gel, Kerr, USA) was applied on the mesial surface for 15 seconds, rinsed for 20 seconds, and slightly air-dried. It is advisable to etch a little more surface area (labial) as the exact location of final restoration margin is uncertain [9]. Then, two coats of a single bottle bonding agent (Adper Single Bond, 3M ESPE, USA) were applied using applicator tips and polymerized for 20 seconds with an LED light (Elipar™ 2500, 3M ESPE Dental products, US). Care was taken to apply uniform coats of the bonding agent especially near the gingival area. Since pooling of the bonding agent compromises solvent evaporation, after careful application of the bonding agent near the sulcus, it was air-thinned using oil-free syringe.

**Figure 4:**
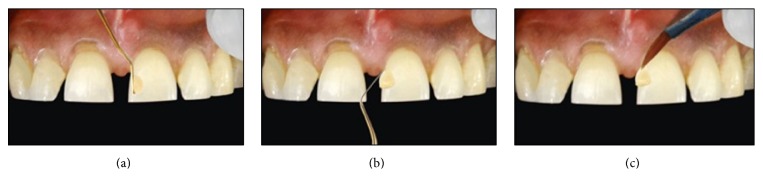
(a) A small increment of composite is taken. (b) This increment is flattened with the instrument and is sculpted towards free gingival margin. (c) Composite is then thinned with a brush to achieve an imperceptible margin.

**Figure 5:**
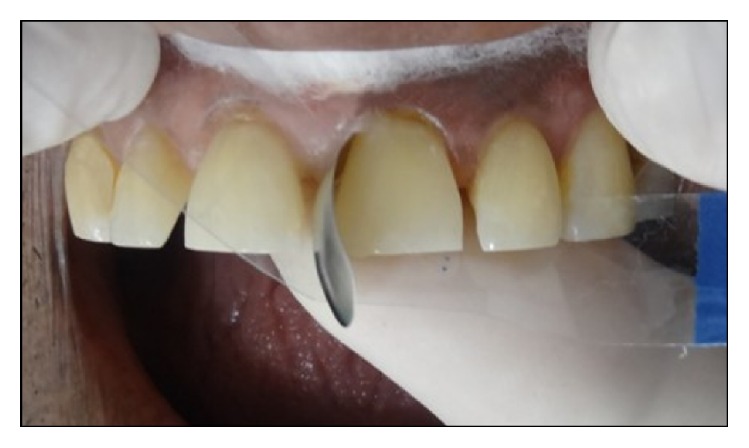
Palodent matrix and mylar strip were applied for restoration of 21.

Following this, a small increment was placed near the “future” contact area and manually contoured over the mesial surface using a long bladed titanium instrument (Figures 4(a) and 4(b)). The composite was then sculpted beneath the free gingival margin and shaped to ideal contours. A brush was then used to thin the material to obtain an imperceptible margin (Figure 4(c)). The increment was cured with LED light for 40 seconds, both from labial and palatal aspects. Then, a contoured sectional matrix (Palodent® System, DENTSPLY Caulk, Milford, Delaware, US) was placed on the mesial surface of 21 with one end slightly into the sulcus (Figure 5). This is to assure the progressive emergence profile of the resin composite. This contoured matrix was then stabilized by holding it from the palatal side and resin composite was then added incrementally to complete the build-up of 21. These contoured matrices are much rigid (unlike mylar strip) which confers them some degree of self-stability. A mylar strip can be placed on the palatal aspect to act as a frame against which to pack composite (Figure 5). Although, some clinicians prefer a gloved finger for this purpose. Each increment was cured for 40 seconds from both labial and palatal aspects. An ET 9 bur (Brasseler, USA) was used to contour and finish the restoration margins. It is recommended to slightly overbuild the first tooth, so that, after finishing and polishing, the tooth achieves the correct mesiodistal dimension.

**Figure 6:**
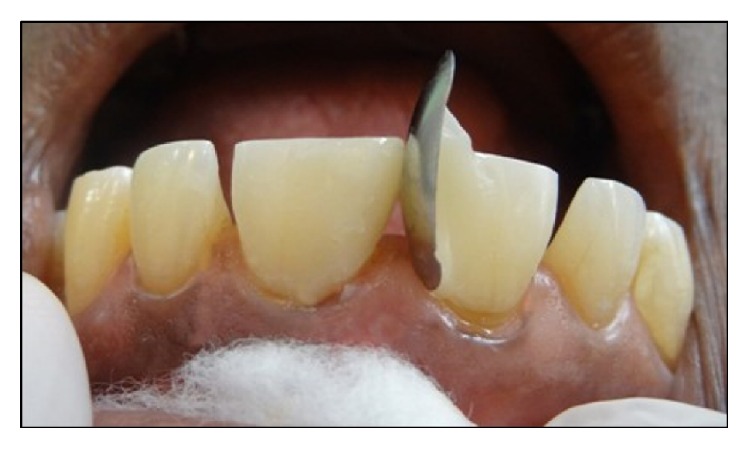
Restoration of 11-matrix should be inserted slightly into the sulcus for correct emergence profile of the composite.

**Figure 7:**
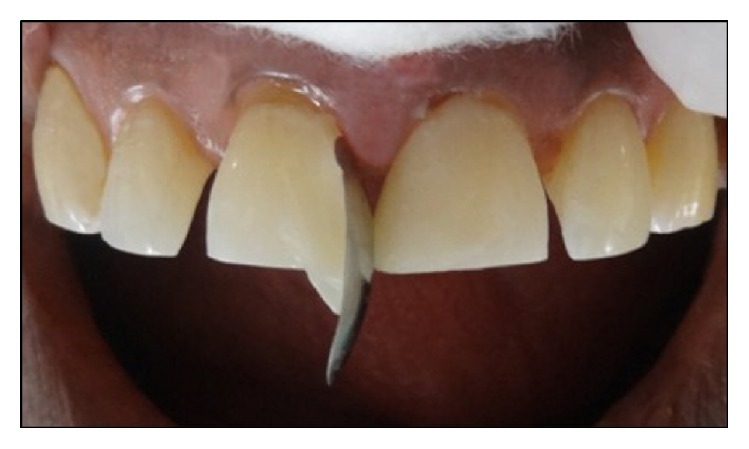
Anatomic contours can be easily achieved using the contoured matrix.

**Figure 8:**
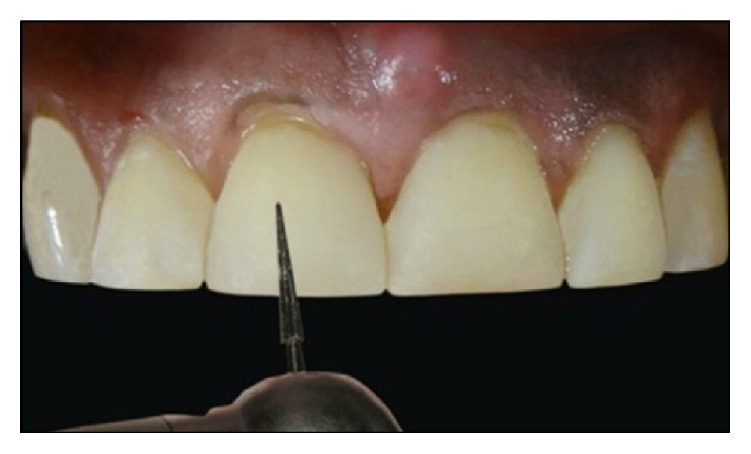
Finishing is done with an ET 9 bur.

**Figure 9:**
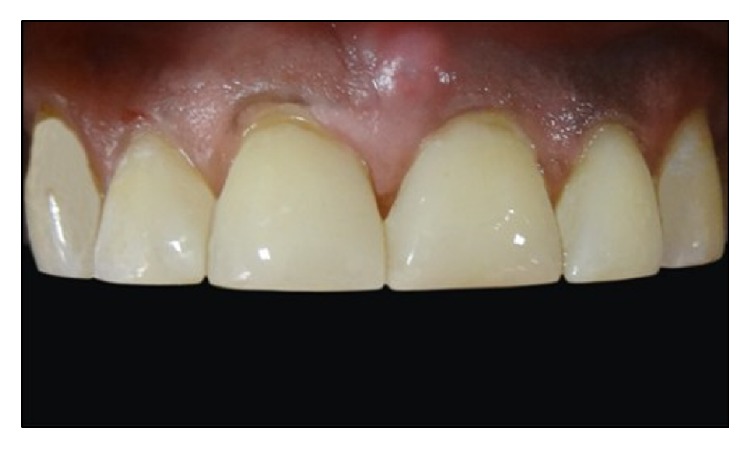
Labial view of upper anterior regions after diastema closure.

**Figure 10 fig10:**
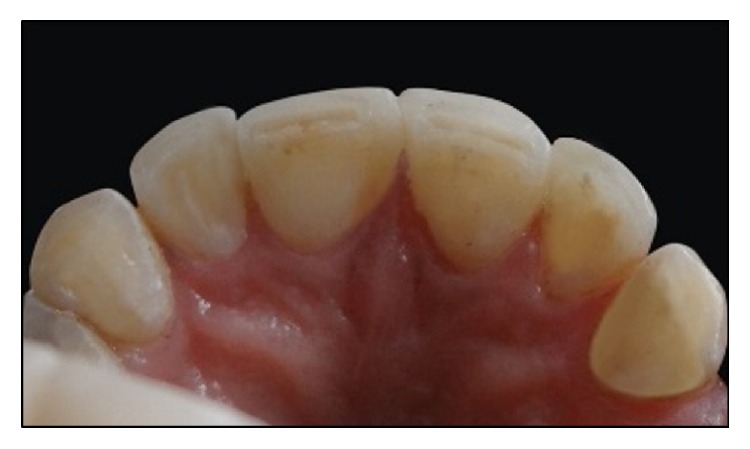
Occlusal view after diastema closure.

**Figure 11:**
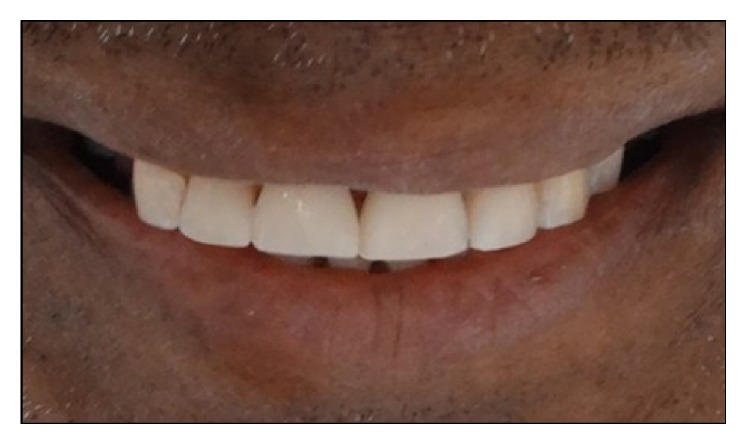
Postoperative extraoral view of the patient.

The same procedure was repeated for 11 (Figures 6 and 7). Care should be taken to place the matrix slightly into the sulcus (Figure 6). Placing and stabilizing sectional matrix in 11 would be much simpler as the mesial surface of 21 would provide it with a “positive stop.” Once the midline diastema was restored, the diastema between central and lateral incisors was closed in the same manner. Once the diastema closure was accomplished in all the teeth, an ET 9 bur was used again for finishing procedure (Figure 8). Final finishing and polishing were accomplished with Sof-Lex discs (3M ESPE Dental Products. St. Paul, MN, USA). The incisal embrasures were kept small and general anatomic forms of teeth were kept flat and broad which best suited his face and body type (see flat canines of the patient). No or minimal characterization was given on the labial aspects of the teeth. Final outcome of the restorative procedure can be seen in Figures 9 [Fig fig10]–11.

Once all the restorations were placed, the occlusion was verified in both centric and eccentric relations using an articulating paper.

**Figure 12:**
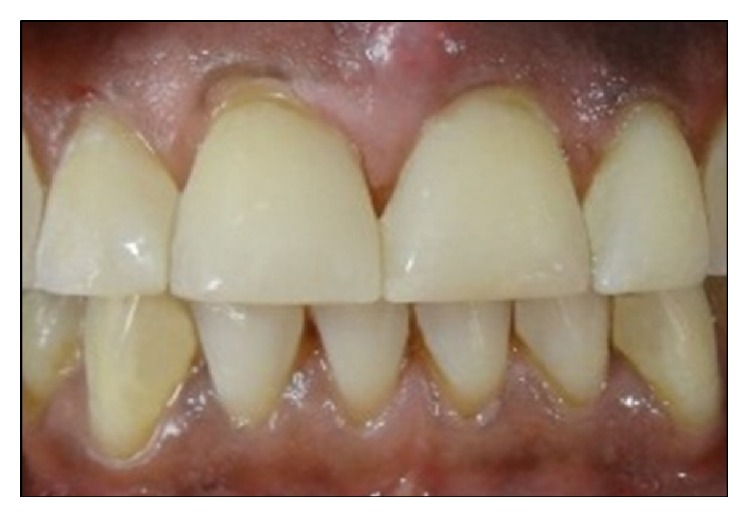
View of the restorations at 14-month recall.

The patient was motivated for oral hygiene and informed for recalls. After 6 months, the restorations were only polished using Sof-Lex discs. The patient was recalled after another 6 months. However, he could not return until 8 months. At 14-month recall (Figure 12), the restorations were evaluated according to modified United States Public Health Service (USPHS) criteria [10]. The scores for all the test procedures were found to be A (Alpha).

## 3. Discussion

Resin-based composite restorations are single-visit procedures and bypass laboratory work which reduces cost of the treatment. They usually do not require wax-ups and preliminary models. In addition to this, some added advantages that these restorations have over other common treatment modalities are that (a) they are gentle towards the opposing dentition, unlike ceramic materials and (b) they are easy to repair in case of fracture. With porcelain restorations, any modification means a return-trip to the laboratory for correction [11, 12].

However, there are some distinct disadvantages that these restorations possess which makes case selection critical. Composite restorations possess less color stability compared to ceramics. This of course is related to the degree and quality of polishing but also depends on the patient maintenance [13]. Our patient demonstrated good oral hygiene and was given further instructions regarding the same. Secondly, they possess less fracture toughness and compressive and shear strength and hence are not suited for high-stress bearing areas [14].

In spite of these disadvantages, the clinicians have been offered the best quality resin materials today which allow them to yield esthetic, functional, economical, and durable restorations. We chose to close the diastema using composite restorative material because it was the most conservative treatment possible, the patient exhibited good periodontal health, and also the patient was not willing for an expensive treatment. Excellent outcomes have been reported by numerous authors who have used resin composites for diastema closures [4, 15, 16]. Willhite [17] proposed three criteria for successful diastema closure: an increased emergence profile with natural contours at the interface between the gingiva and tooth; a completely closed gingival embrasure (i.e., no black triangle); and a smooth subgingival margin that does not catch on or shred dental floss.

A common technique of restoring diastemas is to make impression of the wax-up model and fabricating a silicon putty-index [18, 19]. However, in this case using another technique using contoured sectional matrices was decided. The mesial surfaces of teeth were restored one by one; that is, the first tooth was finished and polished to completion prior to initiation of the second tooth. This allowed us to precisely duplicate the centrals, resulting in two teeth which were mirror images of each other. Most importantly, mesial anatomic contouring could be easily achieved because of the inherent shape of the matrices. One of the biggest challenges that the clinicians face is the failure to avoid “black triangles” when closing diastemas. The restorative technique described here can be applied with relative ease to avoid the “black triangles.” A similar technique by Gresnigt et al. has been previously used in literature for direct laminate veneers [20].

These matrices are especially useful in cases where large diastemas (3-4 mm) have to be closed using direct composite resin restorations. The matrices used in this case are polished stainless steel matrices intended for single use only. Since they are polished and made of “soft” metal, there is no risk of epithelial damage when passively inserted into the sulcus. The manufactures recommend steam autoclaving the matrices at 134°C for 3 minutes prior to clinical use. Unlike the BiTine® rings which can be autoclaved 700 times, the matrices can be autoclaved only once [21].

The composite resins used for anterior restorations must demonstrate good handling (nonsticky and nonslumping) and aesthetic (polishability) characteristics. Few commercially available resin composites (e.g., Estelite Sigma, Tokuyama [Tokyo, Japan]; Filtek Supreme Ultra, 3M ESPE [St. Paul, MN]; Premise, Kerr [Orange, CA]; Renamel Microfill, Cosmedent [Chicago, IL]) are well suited for this purpose [22]. Also, they should contain a high filler content by volume (>65%) and particle size smaller than 5 *μ*m [23]. In the present case, we used Filtek Supreme XT which has a filler loading of 78.5% by weight and an average filler particle size of 0.6–1.4 *μ*m [24].

Though the technique mentioned in this report is easy to perform, but the creation of correct midline and optimal contact area requires experience and skill. The dentist should be well experienced with both the technique and the restorative material to perform the procedure correctly. Although use of rubber dam is said to be of paramount importance in placing composite restorations, using cotton-roll isolation in this case was decided. This is primarily because of two reasons. Ideally, the midline of teeth should coincide with midline of face and while restoring midline diastema, it becomes difficult to visualize the midline of face with the rubber dam in place [25]. Secondly, if the midline is shifted by 4 mm or less it is hardly perceptible to the naked eye, but if it is tilted mesiodistally by even 1° (i.e., canted midline), it is discernible. In the authors' opinion, without rubber dam, it is easy to circumvent both the above problems. On the other hand, this step in no way should compromise the longevity of the restoration. A follow-up after 14 months shows no evidence of discolorations, fractures, debonding, or sensitivities (Figure 12). Although a 14-month follow-up might not seem long enough, the abovementioned restoration related failures generally manifest within 6 months after treatment [5]. Diastema closure only under cotton-roll isolation has been demonstrated previously as well [9].

Apart from silicon putty-index technique and common indirect restorative therapy like ceramic veneers, an indirect ceramic restoration called the “ceramic fragment” is also a treatment option for these cases. However, being an indirect procedure, it requires at least two appointments [6].

Recent studies have concluded that direct composite restorations can be considered aesthetic, functional, and stable restorations in patients with favorable occlusion. Prabhu et al. [26] conducted a study in which midline diastema closure was done in maxillary and mandibular central incisors in a total of 45 patients. Recall visits were made every 6 months for a period of 60 months. The authors stated that composite restorations exhibited satisfactory survival rates. Similarly, Demirci et al. [27] evaluated direct composite build-ups for space closure after orthodontic treatment for 4 years and concluded that survival rates for the restorations were favorable for the specified period. Taking into account that failures such as discoloration, marginal leakage, fracture, and debonding usually occur within 6 months of the placement of the restoration, these long-term studies seem to be predictable indicators of long- life of composite restorations.

By taking the past and current literature into consideration, an experienced clinician with the required skill, proper technique, and case selection can create aesthetic and long-lasting direct resin composite restorations much to the satisfaction of his patients as with the case presented in this report.
